# Improving counseling effectiveness with virtual counselors through nonverbal compassion involving eye contact, facial mimicry, and head-nodding

**DOI:** 10.1038/s41598-023-51115-y

**Published:** 2024-01-04

**Authors:** Doo Sung Choi, Jongyoul Park, Martin Loeser, Kyoungwon Seo

**Affiliations:** 1 Department of Applied Artificial Intelligence, Seoul National University of Science and Technology, 232 Gongneung-ro, Gongneung-dong, Nowon-gu, Seoul, 01811 Korea; 2 Department of Computer Science, Electrical Engineering and Mechatronics, ZHAW Zurich University of Applied Sciences, Winterthur, Switzerland

**Keywords:** Computer science, Information technology

## Abstract

An effective way to reduce emotional distress is by sharing negative emotions with others. This is why counseling with a virtual counselor is an emerging methodology, where the sharer can consult freely anytime and anywhere without having to fear being judged. To improve counseling effectiveness, most studies so far have focused on designing verbal compassion for virtual counselors. However, recent studies showed that virtual counselors’ nonverbal compassion through eye contact, facial mimicry, and head-nodding also have significant impact on the overall counseling experience. To verify this, we designed the virtual counselor’s nonverbal compassion and examined its effects on counseling effectiveness (i.e., reduce the intensity of anger and improve general affect). A total of 40 participants were recruited from the university community. Participants were then randomly assigned to one of two virtual counselor conditions: a *neutral virtual counselor condition* without nonverbal compassion and a *compassionate virtual counselor condition* with nonverbal compassion (i.e., eye contact, facial mimicry, and head-nodding). Participants shared their anger-inducing episodes with the virtual counselor for an average of 16.30 min. Note that the virtual counselor was operated by the Wizard-of-Oz method without actually being technically implemented. Results showed that counseling with a compassionate virtual counselor reduced the intensity of anger significantly more than counseling with a neutral virtual counselor (*F*(1, 37) = 30.822, *p* < 0.001, *η*_p_^2^ = 0.454). In addition, participants who counseled with a compassionate virtual counselor responded that they experienced higher empathy than those who counseled with a neutral virtual counselor (*p* < 0.001). These findings suggest that nonverbal compassion through eye contact, facial mimicry, and head-nodding of the virtual counselor makes the participants feel more empathy, which contributes to improving the counseling effectiveness by reducing the intensity of anger.

## Introduction

Sharing negative emotions resulting from an anger-inducing episode with others is an effective method for alleviating emotional distress^1,2^. By sharing their experience with someone else, the sharer can view their episode from a more objective perspective^3^, which can lead to a reduction in the intensity of anger^2^ and an improvement in their general affect^1^. However, individuals often struggle to share such episodes due to fear of being judged negatively by others for their personal feelings^4^. They may also fear that those providing counseling will become emotionally exhausted from repeated sessions^5^. Above all, if the sharer does not receive sufficient amount of compassion from others about their experiences, it may exacerbate their anger instead^5^. Therefore, there is a need for new technologies that help sharers safely and freely share their anger-inducing episodes with others.

A potential technology that can aid individuals in freely sharing their anger-inducing episodes without the difficulties mentioned earlier is a *virtual counselor*, an animated computer character that utilizes computer graphics and natural language processing^6,7^. The non-judgmental nature of a virtual counselor eliminates the fear of being judged by the individual sharing their personal episodes^8,9^ Additionally, a virtual counselor can listen to the individual’s anger-inducing episodes anytime and anywhere without becoming emotionally exhausted. Above all, a virtual counselor can provide adaptive counseling by understanding and improving the sharer’s emotion through a real-time evaluation during a counseling session^10^. Research has demonstrated that counseling with a virtual counselor is more effective than other counseling methods, such as participants recording their experiences with a voice recorder or camera^11^ or writing about their anger-inducing episodes^12^. Building on these findings, several virtual counselors have been developed and studied to determine which characteristics of virtual counselors are most successful in improving counseling effectiveness^13^.

Recent studies have shown that among many other characteristics, a virtual counselor’s compassionate attitude is critical to improving counseling effectiveness^14–17^. For example, individuals receiving counseling from a compassionate chatbot perceived more support compared to those receiving information-only counseling from a non-empathetic chatbot^18^. Moreover, this compassionate chatbot was useful in reducing the intensity of anger and improving general affect^19^. It should be noted that these studies mainly focused on the design of verbal compassionate behaviors exhibited by the virtual counselor and its effectiveness^20–22^. It is still unclear whether the virtual counselor’s nonverbal compassion (such as eye contact, facial mimicry, and head-nodding) can improve counseling effectiveness. Grondin and colleagues called for more research to investigate the effects of virtual counselors’ nonverbal compassionate behaviors^23^. Therefore, this paper designed a ‘*compassionate virtual counselor*’ that provides nonverbal compassion to the individual and examined its counseling effectiveness compared to a ‘*neutral virtual counselor*’ without nonverbal compassionate features.

This paper is organized as follows. In "Related work", previous studies on compassionate virtual counselors and methods for providing nonverbal compassion are reviewed. "Materials and methods" describes the materials and methods utilized in this study, such as the participants, the two different virtual counselor conditions, emotional and usability questionnaires to assess counseling effectiveness, experimental procedures, and statistical analysis. The key findings on the effects of virtual counselors’ nonverbal compassion on improving counseling effectiveness are presented in "Results". Finally, "Discussion" provides a summary of the study’s conclusions, theoretical and practical implications for designing compassionate virtual counselors, limitations and suggestions for future research.

## Related work

### Compassionate virtual counselors

Previous studies have explored which appearance of the virtual counselor is best suited to convey compassion to the individual seeking counseling. Through experiments utilizing various forms of virtual counselors, including animals, cartoon characters, and robots, it was discovered that individuals perceived the highest level of compassion when the virtual counselor took on a human-like form^24–26^. Further investigation into the effects of age and gender on human-form virtual counselors revealed that individuals perceived the best first impression when the virtual counselor was a young female^27,28^. Taking these findings into account, the present study designed a virtual counselor in the form of a young female to provide effective compassion.

Recent studies have explored how virtual counselors can be designed to improve counseling effectiveness through the provision of verbal compassionate behaviors^29^. Pauw and colleagues have categorized these verbal compassion into emotional and cognitive support^21^. Emotional support involves verbal interactions that provide comfort, validation, and understanding to the individual sharing their experience, such as “I’m sorry to hear that.” On the other hand, cognitive support refers to verbal interactions that help the individual reevaluate the meaning of their anger-inducing episode, such as “It sounds like you're learning, though.” Pauw and colleagues demonstrated that both emotional and cognitive support were effective in reducing the intensity of anger in individuals seeking counseling^21^. Interestingly, the individuals preferred emotional support over cognitive support^21^. Reis and colleagues found that the preference for emotional support could be attributed to the individual’s desire to be understood^30^. Based on these findings, the current study has designed a virtual counselor to provide emotional support when individuals share their anger-inducing experiences.

More recently, Wang and Ruiz have emphasized the importance of researching nonverbal compassion in addition to verbal compassion^31^. Felnhofer and colleagues demonstrated that the stress-buffering effects for individuals seeking counseling can be improved through the provision of nonverbal compassion by virtual counselors^13^. Additionally, Looije and colleagues reported that individuals were more likely to trust virtual counselors who provided not only verbal compassion but also nonverbal compassion^32^. Despite the significance of a virtual counselor’s nonverbal compassion, there is still a lack of clarity regarding how to design such nonverbal compassion. Furthermore, the effects of a virtual counselor’s nonverbal compassion on improving counseling effectiveness, such as reducing the intensity of anger and improving general affect, remain an open question. Therefore, in this study, the nonverbal compassion shown by the virtual counselor is based on how human counselors express nonverbal compassion. We evaluate if and how the additional nonverbal displays of compassion improves the counseling effectiveness.

### Nonverbal compassion

This study has analyzed three types of nonverbal compassion techniques used by human counselors that are known to be effective in improving counseling: *eye contact*, *facial mimicry*, and *head-nodding*. Firstly, eye contact is an important nonverbal compassion technique in forming a positive rapport between the counselor and the individual seeking counseling^33^. Eye contact helps individuals seeking counseling feel that the counselor is ready to listen^34^, and increased eye contact during counseling can enhance trust in the counselor^35^. Specifically, studies have found that counseling was most effective when the duration of the counselor’s eye contact was between 3 to 9 s^35,36^.

Secondly, facial mimicry is a nonverbal compassion technique where the counselor makes facial expressions that correspond to the sharer’s emotions, rather than just imitating the sharer’s expression^37–39^. Appropriate facial mimicry can make the sharer feel that the counselor empathizes with their emotions^40,41^, and increase satisfaction with the counseling experience^42^. Human counselors who are skilled at facial mimicry frown when sharers talk about their anger-inducing episodes and smile when sharers talk about their everyday or positive experiences^43^.

Finally, head-nodding is a nonverbal compassion technique that effectively conveys the counselor’s understanding of the sharer’s feelings^44,45^. Recent studies^46,47^ found that when counselors nod their heads while smiling or blinking, they can better build rapport with sharers. Poppe and colleagues found that nodding 6 to 12 times per minute increased sharers’ satisfaction with their counseling experience^48^. Table 1 summarizes these nonverbal compassion techniques that our virtual counselor can provide.

**Table 1:** The virtual counselor’s nonverbal compassion techniques and descriptions.

Nonverbal compassion technique	Description
Eye contact	Maintain eye contact with the sharer for a duration of 3 to 9 s when the sharer shares their episodes
Facial mimicry	Display empathetic facial expressions that correspond to the sharer’s feelings by frowning when they share anger-inducing episodes and smiling when they share ordinary or pleasant episodes
Head-nodding	Perform head-nodding 6 to 12 times per minute to convey understanding of the sharer’s emotions while attentively listening to their episodes

Many studies on the effects of nonverbal compassion through eye contact, facial mimicry, and head-nodding have been conducted with human counselors, but few studies have been conducted with virtual counselors. To demonstrate the effects of a virtual counselors’ nonverbal compassion on counseling effectiveness, this study designed and compared two different conditions of virtual counselors: *a neutral virtual counselor condition* without nonverbal compassion and a *compassionate virtual counselor condition* with nonverbal compassion. We hypothesized that the virtual counselor’s nonverbal compassion through eye contact, facial mimicry, and head-nodding would improve the effectiveness of counseling with the sharer (i.e., reduce the intensity of anger and improve general affect).

## Materials and methods

### Participants

A total of 40 participants (20 males and 20 females; average age of 22.47 ± 2.69 years) were recruited from the university community. Only participants who were willing to share their private anger-inducing episodes with a virtual counselor were included in the experiment. Informed consent was obtained from all participants after fully explaining experimental procedures. The study protocol was approved by the Institutional Review Board of Hanyang University Hospital (HYUH-2021-08-020-004). All methods were performed in accordance with the Declaration of Helsinki.

### Two different virtual counselor conditions

**Figure 1:**
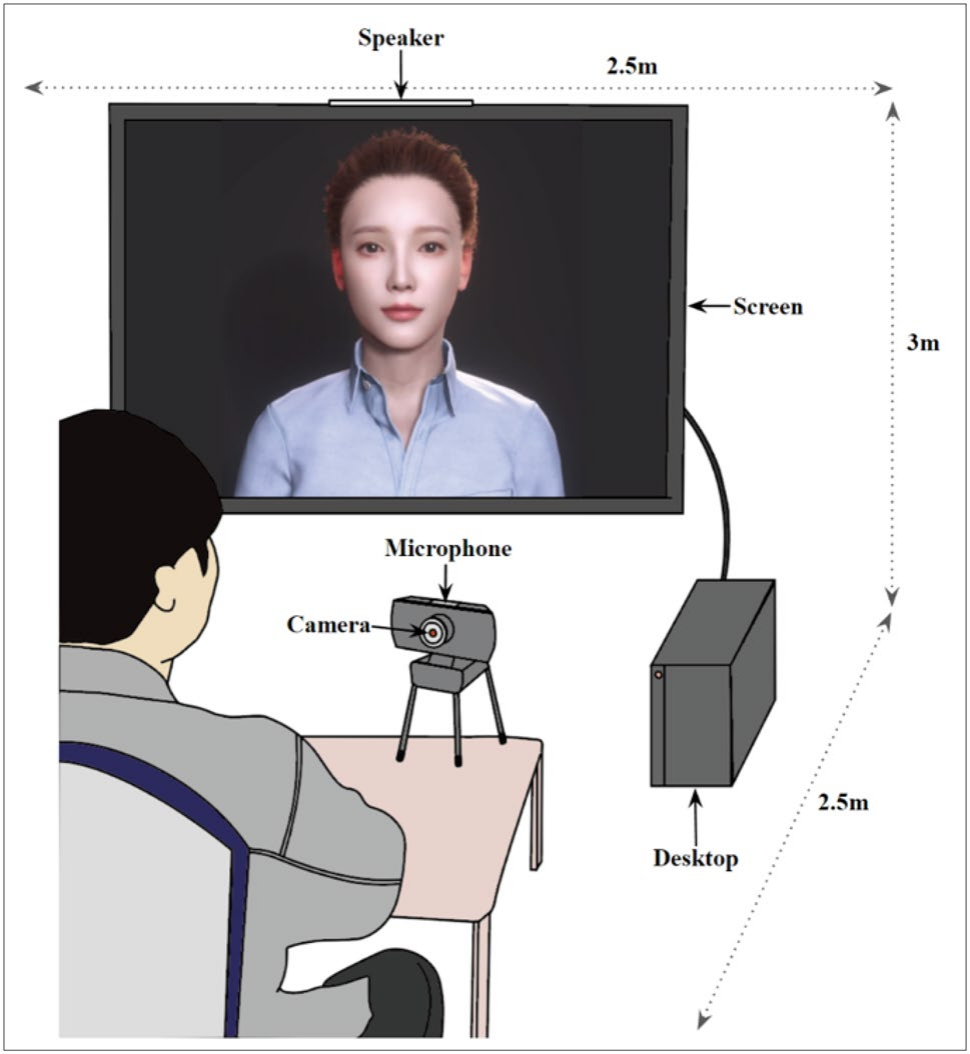
The experimental setup for participants to conduct counseling with a virtual counselor.

In the experimental setup depicted in Fig. 1, participants were able to see the virtual counselor on a 75-inch screen, hear the virtual counselor’s voice through a speaker, and interact with the virtual counselor through a camera and microphone. To protect their privacy, each participant entered a private room individually for counseling with the virtual counselor. The virtual counselor was operated by a human experimenter through the Wizard-of-Oz method, which is an experimental technique used in human–computer interaction studies without real technical implementation^49^. The experimenter (i.e., wizard) observed participants’ behavior through a camera and microphone and managed the virtual counselor based on a predetermined protocol (as detailed in "Experimental procedures"). Initially, participants were instructed to establish and maintain eye contact with the virtual counselor, but this requirement was relaxed once the experiment commenced to avoid potential disruptions. When participants shared their anger-inducing episodes, the experimenter presented a pre-recorded video of the virtual counselor displaying nonverbal compassion, with limited opportunities for the experimenter to naturally establish empathy with the participants. Efforts were made to maintain consistency in the virtual counselor’s verbal aspects, including tone of speech and speech rate, using pre-recorded voice data, to eliminate potential influences from verbal factors. During the experiment, participants implicitly believed that the virtual counselor was an animated computer character with natural language processing and image processing technology. After the experiment concluded, the experimenter disclosed the true operation of the virtual counselor and conducted direct inquiries with participants to assess any privacy concerns. Notably, none of the participants reported privacy violations due to the use of the Wizard-of-Oz method, likely because we deliberately selected participants comfortable with sharing their anger-inducing episodes with others. Additionally, we refrained from recording the Wizard-of-Oz method for privacy reasons. In line with previous studies^28,50^, the virtual counselor was designed as a young woman, as it gave a positive first impression to participants. A high-performance desktop (Intel i7-10700, NVIDIA GeForce RTX 3070, 32 GB RAM) and Reallusion’s software (Character Creator 4 and iClone 8) were used to implement the visual image of the virtual counselor. The image source for the virtual counselor’s face was derived from the package provided by Character Creator 4.

**Figure 2:**
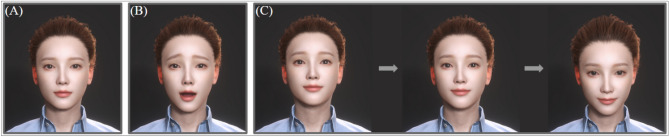
Appearance of nonverbal compassion in the compassionate virtual counselor condition. (**A**) Consistent eye contact of 3 to 9 s when participants share their episodes. (**B**) Facial expressions that correspond to the emotion of the episode shared by the participants, such as frowning or smiling. (**C**) Head-nodding 6 to 12 times per minute while actively listening to participants.

This study designed two different virtual counselor conditions based on the nonverbal compassion techniques presented in Table 1. The first condition was the *neutral virtual counselor condition*, where the virtual counselor did not provide any nonverbal compassion through eye contact, facial mimicry, or head-nodding. The neutral virtual counselor did not maintain eye contact with participants, made random facial expressions, and did not nod their heads well. In contrast, the *compassionate virtual counselor condition* was designed to provide nonverbal compassion to the participants. The virtual counselor in this condition repeated eye contact with the participants for 3 to 9 s when they shared their episodes, frowned or smiled depending on the emotion of the episode shared by the participants, and nodded their heads 6 to 12 times per minute while listening to the participants. The design of the virtual counselor in the compassionate condition was based on previous studies suggesting that these nonverbal compassion techniques are effective in improving counseling effectiveness^36,43,48^. Figure 2 shows an example of the compassionate virtual counselor providing nonverbal compassion to the participant during the experiment. Note that the image source for the virtual counselor’s face originates from the package provided by Character Creator 4.

### Emotional and usability questionnaires

For this study, two questionnaires were used to compare the effectiveness of counseling between two different virtual counselor conditions: *emotional* and *usability* questionnaires. The emotional questionnaire assessed the participants’ intensity of anger and general affect before and after counseling, using a 100-point Likert scale (0 = not at all, 100 = very much; see Supplementary Material S1). The purpose of this questionnaire was to determine whether counseling with a virtual counselor reduced the intensity of anger and improved the general affect of participants regarding the anger-inducing episodes they shared. The usability questionnaire assessed the participants’ satisfaction with counseling with the virtual counselor on a 7-point Likert scale (1 = not at all, 7 = very much) based on five aspects: (1) whether the virtual counselor was good for empathy, (2) whether they found it easy to talk about their personal situations with a virtual counselor, (3) how similar this conversation was to what they normally have in their daily life, (4) how well they thought a virtual counselor did as a conversation partner, and (5) how connected they felt to a virtual counselor after each conversation. All items of the emotional and usability questionnaires were adapted from Pauw et al.^21^.

### Experimental procedures

Participants were randomly assigned to one of two virtual counselor conditions: a neutral virtual counselor or a compassionate virtual counselor. They were then instructed to recall an episode with a family member or close friend that still made them angry. Before counseling with the virtual counselor, participants rated the intensity of their anger and general affect when recalling the anger-inducing episode using an emotional questionnaire. Afterward, participants proceeded with counseling about their anger-inducing episode with a virtual counselor through a six-step process: (Step 1) The virtual counselor built rapport with the participant by asking warm-up questions such as “Did you have any trouble getting here?”, (Step 2) The virtual counselor asked the participant to share details about the anger-inducing episode they experienced by asking questions such as “If you don’t mind, could you tell me about an experience that made you angry?”, (Step 3) The virtual counselor listened to the anger-inducing episode shared by the participant and provided emotional support, such as “It is understandable you feel angry”, (Step 4) The virtual counselor asked additional questions to encourage the participant to talk more about the anger-inducing episode, such as “Which part particularly upset you?” or “Could you elaborate a bit more on your angry feelings?”, (Step 5) The virtual counselor offered emotional support to the participant, such as “I’m sorry to hear that” or “You must be really angry and upset”, and (Step 6) The virtual counselor asked if the participant wanted to say anything more about their anger-inducing episode (e.g., “Is there anything else you'd like to say about your episodes?”) and then concluded the counseling by saying, “Thank you for sharing your episodes today.” During this six-step process, a compassionate virtual counselor provided nonverbal compassion through eye contact, facial mimicry, and head-nodding; whereas a neutral virtual counselor provided no nonverbal compassion. After the counseling, participants re-rated their intensity of anger and general affect when recalling the anger-inducing episode using an emotional questionnaire. They also rated their counseling experience with a virtual counselor using a usability questionnaire and were given the opportunity to provide comments about their overall experience with the virtual counselor. The total duration of the experiment was an average of 16.30 ± 5.01 min. Participants were free to stop the experiment at any time if they felt uncomfortable talking to the virtual counselor, though none chose to do so.

### Statistical analysis

We conducted statistical analysis using IBM SPSS Statistics 27 software. Firstly, we used a χ^2^-test and an independent samples *t*-test to compare basic demographic characteristics, such as gender and age, between participants in the neutral virtual counselor condition and the compassionate virtual counselor condition. Secondly, we used independent samples *t*-tests to compare the intensity of target emotions (i.e., anger and general affect) before counseling between the two virtual counselor conditions, ensuring that participants started with a similar level of emotions. We then conducted a one-way analysis of covariance (ANCOVA) to compare the intensity of target emotions after counseling between the two virtual counselor conditions. The results of the emotional questionnaire before counseling were used as a covariate (i.e., ‘Before counseling’ variable in Table 2), the virtual counselor condition was used as the independent variable, and the emotional questionnaire results after counseling were used as the dependent variable. We then conducted post-hoc analysis using paired-sample *t*-tests to compare the emotional questionnaire results before and after counseling. Thirdly, we used independent samples *t*-tests to compare the usability questionnaire results between participants of each virtual counselor condition. Finally, we conducted a Pearson correlation analysis to examine the relationship between participants’ usability with the virtual counselor and counseling effectiveness, which was measured by the degree to which the intensity of anger decreased after counseling compared to before counseling.

**Table 2:** ANCOVA results of the emotional questionnaire results after counseling.

Variable	df	SS	MS	*F* value	*P* value	*η*_p_^2^
The intensity of anger						
Before counseling	1	4476.729	4476.729	35.106	*p* < 0.001***	0.487
Condition	1	3930.431	3930.431	30.822	*p* < 0.001***	0.454
Error	37	4718.221	127.519			
The intensity of general affect						
Before counseling	1	3441.387	3441.387	24.731	*p* < 0.001***	0.401
Condition	1	209.433	209.433	1.505	0.228	0.039
Error	37	5148.613	139.152			

*df* degrees of freedom, *SS* sum of squares, *MS* mean square, *η*_*p*_^*2*^ partial eta squared.

*p < 0.05; **p < 0.01; ***p < 0.001.

## Results

### Basic demographic characteristics

A total of 40 participants were randomly assigned to either the neutral virtual counselor condition (n = 20; 10 males; average age 22.11 ± 1.21 years) or the compassionate virtual counselor condition (n = 20; 10 males; average age 22.66 ± 1.41 years). Basic demographic characteristics, including gender and age, were compared between the two virtual counselor conditions using a χ^2^-test and an independent samples *t*-test. The results indicated that there was no significant difference in gender (χ^2^(1) = 0.100, *p* = 0.752) or age (*t*(38) = -1.217, *p* = 0.845) between the two conditions.

### Emotional questionnaire results

The intensity of anger and general affect before counseling between the two virtual counselor conditions was compared using independent samples *t*-tests. The results revealed no significant difference in the intensity of anger before counseling (*t*(38) = −1.002, *p* = 0.494) or the intensity of general affect (*t*(38) =  −0.469, *p* = 0.334) between the two conditions. An ANCOVA was used to analyze the emotional questionnaire results of the intensity of anger and general affect. Table 2 shows that the intensity of anger before counseling was significantly related to the intensity of anger after counseling (*F*(1, 37) = 32.539, *p* < 0.001, *η*_p_^2^ = 0.638), so it was used as a covariate (i.e., ‘Before counseling’ variable in Table 2). The results indicated that there was a significant effect of condition on the intensity of anger after counseling (*F*(1, 37) = 30.822, *p* < 0.001, *η*_p_^2^ = 0.454), with participants in the compassionate virtual counselor condition showing a significantly greater reduction in intensity of anger (35.000 ± 17.770 points) than those in the neutral virtual counselor condition (54.550 ± 12.967 points). In case of the intensity of general affect, although the intensity of general affect before counseling was significantly related to the intensity of general affect after counseling (*F*(1, 37) = 24.731, *p* < 0.001, *η*_p_^2^ = 0.401), there was no significant effect of condition on the intensity of general affect after counseling (*F*(1, 37) = 1.505, *p* = 0.228, *η*_p_^2^ = 0.039). Overall, the intensity of general affect was generally high for both conditions (70.500 ± 16.376 points for the compassionate virtual counselor condition and 64.500 ± 13.563 points for the neutral virtual counselor condition), consistent with previous studies^21^.

**Figure 3:**
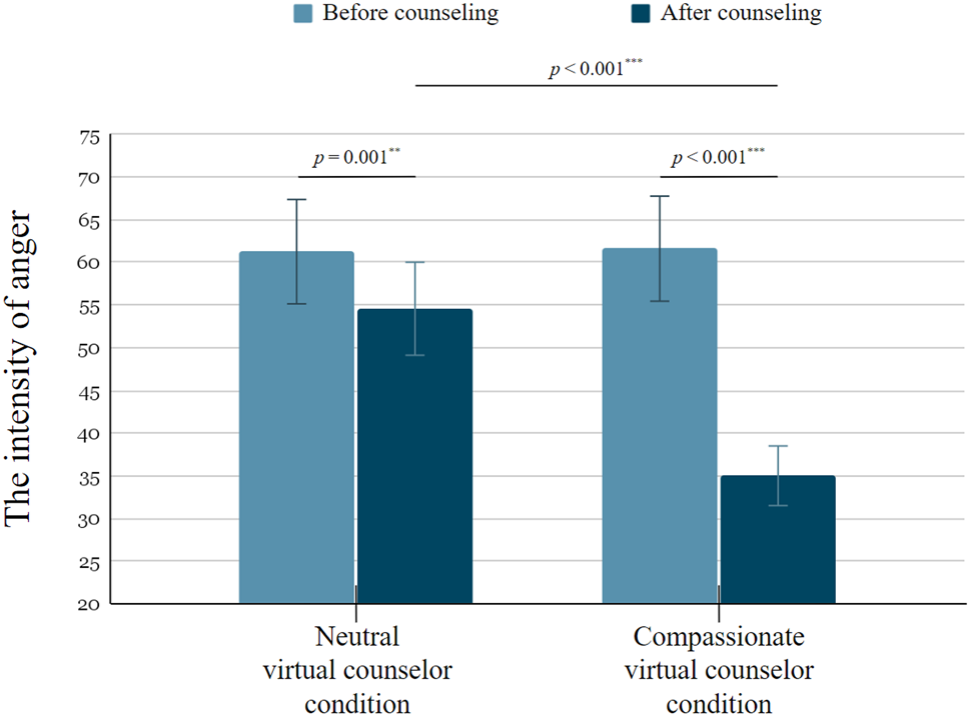
Comparison of the intensity of anger before and after counseling with the neutral virtual counselor condition and compassionate virtual counselor condition.

The emotional questionnaire results before and after counseling were compared using post-hoc analysis with paired-sample *t*-tests. Since the ANCOVA results only showed a significant difference in the intensity of anger after counseling, the post-hoc analysis was only conducted on this variable. As illustrated in Fig. 3, the paired-sample *t*-tests indicated a statistically significant reduction in the intensity of anger before and after counseling in both the neutral virtual counselor condition (*t*(19) = 4.159, *p* = 0.001, effect size* d* = 1.610) and the compassionate virtual counselor condition (*t*(19) = 8.155, *p* < 0.001, effect size *d* = 3.261). Taken together, the ANCOVA and subsequent post-hoc analyses indicate that both virtual counselor conditions resulted in a reduction of anger intensity, but the compassionate virtual counselor was found to be more effective.

### Usability questionnaire results

Table 3 presents the results of the usability questionnaire, indicating that participants perceived significantly more empathy from the compassionate virtual counselor than the neutral virtual counselor (*t*(38) =  −6.179, *p* < 0.001). However, there were no significant differences between the two conditions in other usability aspects, including ease of talking about personal situations (*t*(38) =  −0.104, *p* = 0.918), similarity to daily conversations (*t*(38) =  −1.106, *p* = 0.276), perception of the virtual counselor as a good conversation partner (*t*(38) =  −1.542, *p* = 0.131), and feeling of intimacy with the virtual counselor (*t*(38) =  −1.043, *p* = 0.304).

**Table 3:** Usability questionnaire results after counseling.

	Neutral virtual counselor condition	Compassionate virtual counselor condition	*P* value
Whether the virtual counselor was good for empathy	2.600 ± 0.940	5.050 ± 1.504	*p* < 0.001***
Whether they found it easy to talk about their personal situations with a virtual counselor	3.550 ± 1.234	3.600 ± 1.759	0.918
How similar this conversation was to what they normally have in their daily life	3.800 ± 1.361	4.250 ± 1.209	0.276
How well they thought the virtual counselor did as a conversation partner	4.550 ± 1.317	5.150 ± 1.137	0.131
How connected they felt to a virtual counselor after each conversation	4.300 ± 1.689	4.800 ± 1.322	0.304

Values are expressed as mean ± SD.

*p < 0.05; **p < 0.01; ***p < 0.001.

### Correlation analysis results

**Figure 4:**
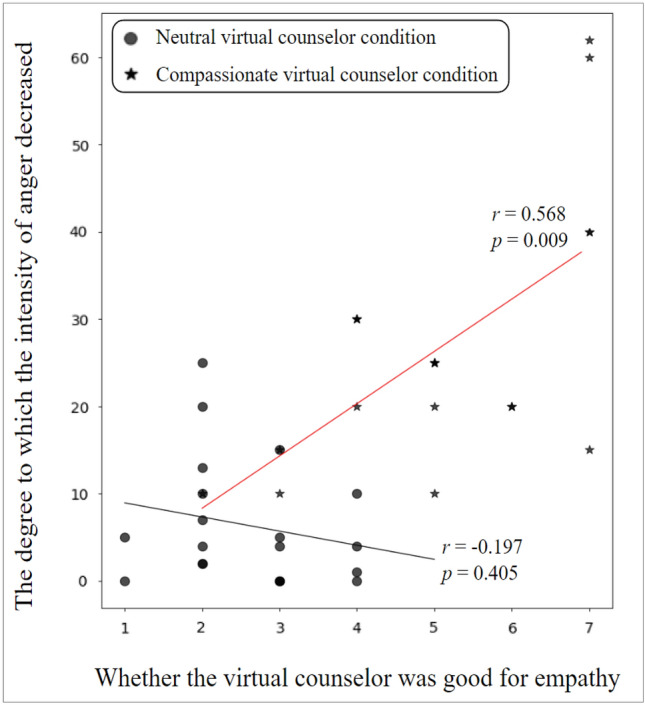
Pearson correlation analysis between usability questionnaire results (*inter alia*, whether the virtual counselor was good for empathy) and emotional questionnaire results (*inter alia*, the degree to which the intensity of anger decreased after counseling compared to before counseling) for neutral virtual counselor condition and compassionate virtual counselor condition.

To examine the impact of compassion on counseling effectiveness, Pearson correlation analysis was conducted between usability questionnaire results (*inter alia*, whether the virtual counselor was good for empathy) and emotional questionnaire results (*inter alia*, the degree to which the intensity of anger decreased after counseling compared to before counseling). As shown in Fig. 4, participants who received counseling from a compassionate virtual counselor exhibited a statistically significant correlation between their perceived level of empathy from the virtual counselor and the degree to which their anger intensity decreased (*r* = 0.568, *p* = 0.009). However, this correlation was not observed in participants who received counseling from a neutral virtual counselor (*r* = −0.197,* p* = 0.405). These findings replicate prior research demonstrating that participants’ perception of empathy is positively related to the effectiveness of counseling^30,51^. Interestingly, while the data revealed a significant linear correlation, a unique observation was made with four participants who rated the perceived level of empathy from the compassionate virtual counselor at 7 points. They showed notable variance in anger intensity reduction. Our anecdotal interview suggested that this variance stemmed from the participants’ preferences for the type of verbal compassion they desired from the virtual counselor (i.e., emotional or cognitive support). For instance, participants #3, #11, and #23, who preferred emotional support, not only perceived a high level of empathy but also experienced a substantial decrease in the intensity of anger after counseling. Conversely, participant #28, with a preference for cognitive support, perceived a high level of empathy from the virtual counselor but sought more practical cognitive support rather than emotional assistance. This preference led to a relatively slight decrease in anger intensity after counseling for participant #28, especially when compared to participants #3, #11, and #23.

## Discussion

The primary goal of this study was to investigate how a virtual counselor’s nonverbal compassion (such as eye contact, facial mimicry, and head-nodding) enhances counseling effectiveness. To compare the effectiveness of counseling, we developed two virtual counselor conditions, the ‘*neutral virtual counselor condition*’ without nonverbal compassion, and the ‘*compassionate virtual counselor condition*’ with nonverbal compassion. Consistent with our hypothesis, counseling with a compassionate virtual counselor condition was more effective in reducing the intensity of participants’ anger than counseling with a neutral virtual counselor condition. Both the compassionate and neutral virtual counselor conditions were effective in maintaining a positive state for participants’ general affect. The correlation analysis revealed a positive relationship between the degree of perceived empathy from the virtual counselor and counseling effectiveness, but only for those who counseled with a compassionate virtual counselor. Overall, our results demonstrate that nonverbal compassion (i.e., eye contact, facial mimicry, and head-nodding) of virtual counselors has a positive impact on counseling effectiveness.

The results of this study provide interesting findings about the process in which the virtual counselor’s nonverbal compassion affects the counseling effectiveness. First, it seems that the virtual counselor’s nonverbal compassion effectively enhanced the sense of empathy experienced by the participants (see Supplementary Table S1 for a compilation of participants’ responses). After the experiment, participants who received counseling from a compassionate virtual counselor reported the following: “The virtual counselor’s eye contact made me feel that they were genuinely attentive to my anger-inducing experiences, leading me to perceive empathy from the virtual counselor” (participant #3), “The virtual counselor’s facial mimicry, mirroring my emotions, made me feel that they were empathizing with my anger-inducing episodes” (participant #7), and “The virtual counselor's head-nodding validated my opinions and instilled a sense of empathy from the virtual counselor” (participant #11). These remarks indicate that the participants perceived a high level of empathy from the virtual counselor thanks to the nonverbal compassion displayed by the virtual counselor. Research conducted by Kemper and Shaltout^52^ proposed the idea that nonverbal expressions of compassion can yield psychophysiological benefits. In other words, nonverbal compassion such as eye contact, facial mimicry, and nodding from the virtual counselor are important factors that have a positive impact on the counseling experience.

Second, the greater the level of compassion participants perceived from the virtual counselor, the more notable the decrease in anger emotions observed following the counseling session. This phenomenon can be explained by the *therapeutic alliance*, which is a partnership between a participant and his or her counselor that allows them to achieve goals through agreed-upon tasks^53^. Moyers and Miller showed that the therapeutic alliance is an essential element for successful counseling effectiveness^54^. The weakened therapeutic alliance in counseling with a counselor can result in poorer counseling effectiveness, and as a result, counseling about angry episodes may hardly reduce the intensity of anger. This weakened therapeutic alliance is well depicted by comments from participants who counseled with a neutral virtual counselor: “My anger did not decrease significantly after the consultation because I did not feel connected to the virtual counselor” (participant #6, #10, and #16). In summary, it can be said that the nonverbal compassion shown by the virtual counselor makes the subject feel greater empathy, and this wins in improving the counseling effect by establishing a therapeutic alliance.

Third, the virtual counselor’s nonverbal compassion can even change the impression of verbal interactions. Despite providing the same verbal interactions with the same voice in both virtual counselor conditions, participants in each condition perceived the virtual counselor’s voice differently after counseling. For instance, a participant (#12) who counseled with the neutral virtual counselor replied, “When the virtual counselor was trying to comfort me, it felt like they were providing insincere verbal interactions and their voice sounded like they were being sarcastic.” On the other hand, another participant (#9) who counseled with the compassionate virtual counselor replied, “I felt that the virtual counselor’s verbal interactions were sincere, and their soothing voice helped me feel at ease.” These results could be explained by LeBlanc and colleagues’ findings that people’s perceptions are influenced by the emotions they experienced at the time they want to recall^55^. In other words, a weakened therapeutic alliance in counseling with a neutral virtual counselor due to the absence of the virtual counselor’s nonverbal compassion may have formed negative emotions toward the virtual counselor during counseling, which could have negatively influenced the perception of the virtual counselor’s voice.

Taken together, our study reveals a potential process by which a virtual counselors’ nonverbal compassion enhances the effectiveness of counseling. When participants perceived a high level of compassion from the compassionate virtual counselor, a therapeutic alliance could be formed between the virtual counselor and the participants. This therapeutic alliance contributed to a greater reduction in anger feelings through counseling. Interestingly, when no therapeutic alliance was formed, participants not only did not feel empathy for the virtual counselor, but also perceived the voice of the virtual counselor negatively. In this context, providing nonverbal compassion through a virtual counselor is essential to enhance counseling effectiveness.

### Practical implications

This study offers three practical contributions about how the virtual counselor’s nonverbal compassion (i.e., eye contact, facial mimicry, and head-nodding) should be constructed for improving counseling effectiveness. First, to enhance the counseling effectiveness by increasing trust in the virtual counselor, the virtual counselor ought to ensure frequent eye contact during counseling. As reported by comments provided by participants after counseling, it was revealed that participants trusted the virtual counselor due to compassion induced by the virtual counselor’s frequent eye contact. This finding is consistent with the findings of a prior study^56^, which suggested that if participants perceived compassion from the virtual counselor, it would likely result in increased trust in the virtual counselor. To provide participants with the virtual counselor’s frequent eye contact, eye-tracking technology that records eye movements across time can be used (e.g.^57,58^). For instance, by utilizing eye-tracking technology to analyze participants’ eye movements, the virtual counselor can increase participants’ trust in virtual counselors by aligning their own eye contact with the participant’s gaze.

Second, to improve the counseling effectiveness by increasing the degree to which participants perceived compassion from the virtual counselor, the virtual counselor should express facial mimicry corresponding to participants’ emotions. While participants generally acknowledged that a combination of nonverbal compassion techniques, including eye contact, facial mimicry, and head-nodding, contributed to their sense of compassion and assisted in mitigating their anger, a majority of those who received counseling from a compassionate virtual counselor emphasized the significance of the virtual counselor’s facial mimicry. They expressed sentiments such as: “When I talked about my anger-inducing episodes during counseling, I felt like I was receiving empathy from the virtual counselor due to their frowning expression” (participant #7, #9, and #13). Furthermore, some participants (8 out of 40) specifically noted that they experienced increased empathy through facial mimicry, resulting in a reduction of their anger: “The virtual counselor’s frowning expression helped me alleviate my anger because it conveyed empathy for my emotions” (participant #15, #19, #24, #29, #30, #34, #35, and #39). These findings align with a prior study^40^ that demonstrated how facial mimicry by human counselors enhances participants’ perceived empathy, showing that a similar effect applies to virtual counselors. These positive effects of virtual counselor’s facial mimicry can be attributed to the concept of therapeutic alliance^59^. When the virtual counselor displays compassionate facial expressions, participants feel a stronger connection with them, potentially amplifying the effectiveness of counseling. To achieve this, speech emotion recognition technology, which analyzes sharers’ emotions (e.g., happiness, sadness, anger, and neutral) through their speech^60^, can be used. For instance, the virtual counselor’s facial mimicry can be expressed based on the emotions of the participants, analyzed by speech emotion recognition technology, appearing as a smile during happy episodes and as a sad expression during sad episodes.

Lastly, to improve counseling effectiveness by building rapport with participants, the virtual counselor’s head-nodding should last while participants are telling. In fact, in comments provided by participants after counseling, most participants who counseled with a compassionate virtual counselor said that “It could help in building rapport with the virtual counselor due to the virtual counselor’s constant head-nodding” (participant #1, #19, and #21). These findings are consistent with prior research that found that a virtual counselor’s head-nodding, accompanied by blinking, can be helpful in building rapport^46,47^. To implement the virtual counselor’s head-nodding that lasts while participants are telling, automatic speech recognition technology, which enables machines to understand human language, can be used. For example, the virtual counselor’s constant head-nodding may appear, when the automatic speech recognition technology detects that participants are telling their episodes.

### Limitations and future directions

This study has several limitations. First, we implemented the virtual counselor’s nonverbal compassion with similar meanings in most socio-cultural aspect, but the nuances of nonverbal compassion in specific cultures can be subtly different^61^. Specifically, Argyle and colleagues demonstrated that maintaining eye contact is generally perceived positively by Western Europeans, while it may not hold true in East Asian cultural backgrounds^62^. Future research should aim to incorporate socio-cultural variations when designing virtual counselors’ nonverbal compassionate expressions. Secondly, participants took an average of 16.30 min to disclose their anger-inducing episodes to the virtual counselor. Our findings do not reflect long-term emotional recovery but only relate to self-reported short-term emotional recovery. A recent systematic review^63^ highlights the promising potential of compassionate technology within blended and digital mental health interventions. In this context, future research should examine the long-term emotional recovery of virtual counselors’ nonverbal compassion, to validate whether it effectively enhances counseling outcomes even through repeated counseling. Furthermore, this study concentrated solely on anger as a prominent negative emotion in modern society^64^. While brief counseling can be effective in ameliorating anger, other emotions, such as depression, require long-term emotional recovery strategies. Consequently, future research should explore the long-term effects of virtual counselors’ nonverbal compassion across a broader spectrum of emotions, encompassing not only anger but also depression and various emotional states. Thirdly, we operated the virtual counselor by Wizard-of-Oz method without actually being technically implemented. With the recent advancements in deep learning technology, it is now possible to design virtual counselors that can automatically provide appropriate empathy to participants. In the future, the actual development and validation of automated virtual counselors using deep learning technology will be necessary. Moreover, while our research primarily focused on examining the impact of nonverbal compassion on counseling effectiveness, there is potential value in quantitatively measuring participants’ empathy using tools like the Davis interpersonal reactivity index^65^. This quantitative approach could provide a deeper understanding of participants’ perceptions of empathy. Finally, despite confirming the positive effects of individual nonverbal compassion techniques, including eye contact, facial mimicry, and head-nodding, on counseling effectiveness, questions remain regarding how the synergy among these diverse nonverbal compassion techniques can further reduce anger intensity. As a result, it would be intriguing to explore the influence of interactions among these various nonverbal compassion techniques on counseling effectiveness.

## Conclusion

Overall, this study examines the impact of virtual counselors’ nonverbal compassion (eye contact, facial mimicry, and head-nodding) on counseling effectiveness. Results show that the virtual counselor’s nonverbal compassion reduces anger intensity and increases perceived empathy. Correlation between usability questionnaire results and emotional questionnaire results was observed specifically with compassionate virtual counselors. This suggests that nonverbal compassion, effective in human counseling, also promotes successful counseling with virtual counselors. Our findings further explain the influence of nonverbal compassion on counseling effectiveness through participant-perceived empathy and therapeutic alliance. Also, practical implications include eye-tracking, speech emotion recognition, and automatic speech recognition technologies for tailored nonverbal compassion were proposed for future implementations.

### Supplementary Information

Supplementary Information.

## Data Availability

All data and materials related to the study are securely stored at the Department of Applied Artificial Intelligence, Seoul National University of Science and Technology. Access to this data can be obtained by submitting a request to the corresponding author.
