# Supplementary material for: Improving counseling effectiveness with virtual counselors through nonverbal compassion involving eye contact, facial mimicry, and head-nodding
Source: Sci Rep. 2024 Jan 4;14:506. doi: 10.1038/s41598-023-51115-y (PMC10766597; doi:10.1038/s41598-023-51115-y)
Supplement: Supplementary file 1 — Supplementary Information. [file 41598_2023_51115_MOESM1_ESM.docx]

**Supplementary Information:**

# **Improving counseling effectiveness with virtual counselors through nonverbal empathy involving eye contact, facial mimicry, and head-nodding**

**Doo Sung Choi^a, †^, Jongyoul Park^a, †^, Martin Loeser^b^, Kyoungwon Seo^a, *^**

**^a^** Department of Applied Artificial Intelligence, Seoul National University of Science and Technology, Seoul, Korea, {dschoi, jongyoul, kwseo}@seoultech.ac.kr

**^b^** Department of Computer Science, Electrical Engineering and Mechatronics, ZHAW Zurich University of Applied Sciences, Winterthur, Switzerland, loma@zhaw.ch

**^†^** These authors contributed equally to this work.

**^*^ Correspondence**

Dr. Kyoungwon Seo

232 Gongneung-ro, Gongneung-dong, Nowon-gu, Seoul, Korea, 01811

Tel: +82 2 970 9777

Email: kwseo@seoultech.ac.kr

**Supplementary Material S1.** Emotional questionnaire to assess the intensity of anger and general affect (adapted from Pauw et al., 2022).

- Before counseling with a virtual counselor:
  - “How are you feeling at this moment? Please rate your current intensity of general affect using a scale from 0 (not at all) to 100 (very much).”

|  |
| --- |

- - “Have you experienced a situation where you got angry with a friend or family member recently, and the issue remains unresolved? If so, please describe in detail what made you angry.”

|  |
| --- |

- - “Please rate your current intensity of anger regarding the anger-inducing episode above using a scale from 0 (not at all) to 100 (very much).”

|  |
| --- |

- After counseling with a virtual counselor:
  - “Please rate your current intensity of general affect using a scale from 0 (not at all) to 100 (very much).”

|  |
| --- |

- - “Please rate your current intensity of anger regarding the anger-inducing episode you shared, using a scale from 0 (not at all) to 100 (very much).”

|  |
| --- |

Pauw, L. S., Sauter, D. A., van Kleef, G. A., Lucas, G. M., Gratch, J., & Fischer, A. H. (2022). The avatar will see you now: Support from a virtual human provides socio-emotional benefits. *Computers in Human Behavior*, *136*, 107368.

**Supplementary Table S1**. A complete list of representative comments participants made about each nonverbal empathy technique (including eye contact, facial mimicry, and head-nodding) after counseling in either the neutral virtual counselor condition without nonverbal empathy or the empathic virtual counselor condition with nonverbal empathy

| **Nonverbal empathy technique** | **Neutral**  **virtual counselor condition** | **Empathetic**  **virtual counselor condition** |
| --- | --- | --- |
| Eye contact | “The virtual counselor not making eye contact with me led me to believe that the virtual counselor was not paying attention to me” (participant #2, #5, #14, and #20)  “I sensed that the virtual counselor wasn't attentive to my words due to the lack of eye contact” (participant #22)  “The virtual counselor did not make eye contact with me, which negatively affected my counseling experience” (participant #31)  “I perceived that the virtual counselor didn't fully comprehend my anger-inducing episodes due to the absence of eye contact” (participant #38) | “The virtual counselor’s eye contact made me feel that they were genuinely attentive to my anger-inducing experiences, leading me to perceive empathy from the virtual counselor” (participant #3)  “Due to the virtual counselor’s welcoming eye contact, I believe it contributed to establishing a rapport with the virtual counselor” (participant #4, #18, #23, and #32)  “I had no reservations in sharing my anger-inducing episode with the virtual counselor because the presence of eye contact made me feel a sense of trustworthiness” (participant #27)  “The virtual counselor's attentive gaze while I shared my story motivated me to be more open in my narrative, enabling me to delve deeper into my thoughts and emotions” (participant #34) |
| Facial mimicry | “I didn't feel connected because of the virtual counselor's facial expression, so my anger didn’t decrease much after the session” (participant #6, #10, and #16).  “When the virtual counselor was trying to comfort me, it felt like they were providing insincere verbal empathy and their voice sounded like they were being sarcastic” (participant #12).  “The virtual counselor's facial expression appeared disinterested in my story, making the counseling feel meaningless” (participant #36)  “When I talked about my anger-inducing episodes, the virtual counselor's facial expression was ambiguous, which made me wonder if the virtual counselor was really understanding of my episodes” (participant #40) | “When I talked about my anger-inducing episodes during counseling, I felt like I was receiving empathy from the virtual counselor due to their frowning expression” (participant #7, #9, and #13).  “The virtual counselor's facial mimicry, mirroring my emotions, made me feel that they were empathizing with my anger-inducing episodes” (participant #7)  “The virtual counselor’s frowning expression helped me alleviate my anger because it conveyed empathy for my emotions” (participant #15, #19, #24, #29, #30, #34, #35, and #39)  “I felt that the virtual counselor's verbal empathy was sincere, and their soothing voice helped me feel at ease” (participant #9) |
| Head-  nodding | “I had concerns about the virtual counselor not nodding their head during the consultation” (participant #8, #17, and #25)  “I sensed that the virtual counselor couldn't connect with my emotions due to the lack of head-nodding” (participant #26)  “I believed that the virtual counselor's lack of head-nodding had no impact on the counseling's outcome” (participant #33)  “I had the impression that the virtual counselor didn't grasp my emotions when they refrained from nodding while listening to my anger-inducing episodes” (participant #37) | “It could help in building rapport with the virtual counselor due to the virtual counselor's constant head-nodding” (participant #1, #19, and #21).  “The virtual counselor's head-nodding validated my opinions and instilled a sense of empathy from the virtual counselor” (participant #11).  “The virtual counselor's nodding conveyed a sense of endorsement for my viewpoint” (participant #28)  “As I discussed my anger-inducing episodes, the virtual counselor's nodding made me feel as though an attentive friend was actively listening to my narrative” (participant #35)  “I could place my trust in the virtual counselor, all thanks to their head-nodding, which demonstrated their comprehension of my narrative” (participant #39) |
